# Perceptual Categories Derived from Reid’s “Common Sense” Philosophy

**DOI:** 10.3389/fpsyg.2017.00893

**Published:** 2017-06-06

**Authors:** Adam Reeves, Birgitta Dresp-Langley

**Affiliations:** ^1^Department of Psychology, Northeastern University, BostonMA, United States; ^2^Centre National de la Recherche Scientifique, University of StrasbourgStrasbourg, France

**Keywords:** sensation, perception, imagery, category

## Abstract

The 18th-century Scottish ‘common sense’ philosopher Thomas Reid argued that perception can be distinguished on several dimensions from other categories of experience, such as sensation, illusion, hallucination, mental images, and what he called ‘fancy.’ We extend his approach to eleven mental categories, and discuss how these distinctions, often ignored in the empirical literature, bear on current research. We also score each category on five properties (ones abstracted from Reid) to form a 5 × 11 matrix, and thus can generate statistical measures of their mutual dependencies, a procedure that may have general interest as illustrating what we can call ‘computational philosophy.’

## Introduction

Our task in this paper is to re-introduce to current researchers a logical basis for the distinction between perception and sensation due to the Scottish ‘Common Sense’ philosopher Thomas Reid (1710–1796), and to show how his approach helps us distinguish between other categories of perceptual experience. We say ‘re-introduce’ because recent developments in Neuroscience, admirable, even astonishing, as they are, have sometimes obscured such distinctions. We suggest that this phase in the history of science is temporary, and that insights are to be gained from the older terms, even though they reflect a refined ‘folk’ psychology (Churchland, 1998) or what Reid himself called ‘vulgar’ usage. We also speculate that the perceptual categories may be placed on a continuum of realism and as such, be subject to a computational analysis rather than simply described as same or different on logical grounds, critical as this latter step is.

Though less influential than Locke or Hume, Reid became known in his lifetime for his theory of perception and its wider implications in the epistemology of science. He published important criticisms of the philosophies of Locke, Berkeley and Hume, in particular attacking their doctrines of Ideas^1^ and representation. Very briefly, he viewed ideas and experiences as mental *activities* rather than being private mental *objects* (as in Hume and Berkeley), and instead of deriving perception from sensation, he analyzed perception into conception and belief, claiming that perception was normally, but not always, accompanied by sensation. He argued for nativism and direct perception, as well as contributing to broader philosophical topics in ethics, aesthetics, free will, and the philosophy of mind (Reid, 1764/1977, 1786). Of direct relevance here, he proposed a number of detailed arguments, combined with nuanced reasoning and attention to physiological optics, to describe how the categories of perception arise. Our aim is to characterize these perceptual categories on the basis of Reid’s analysis, but also including mental imagery and (his term) ‘fancy,’ that is, mental contents not connected to reality. Our aim is not to provide a detailed exegesis of Reid or his contributions to philosophy and epistemology, all of which have received detailed scholarly attention (e.g., Lehrer, 1989; Cuneo and van Woudenberg, 2004), or even of Reid’s greatest contribution to science, his astounding discovery of the non-Euclidean (elliptical) nature of visual space, which pre-dated Reiman by 60 years (Reid, 1764/1977, Chapter 5, Section 9; Daniels, 1974) and is well worth study for its own sake. Rather, we aim to improve the discussion and conceptualization of mental categories in current perception research.

## What is ‘Perception’?

According to Reid (1764/1977), perception is knowledge of the external world through direct experience, as mediated by a sense such as vision, hearing, smell, or touch (all of which he considers). By ‘knowledge’ is meant veridicality; that is, if I perceive an apple before me, then there must be an apple. The everyday terms ‘see’ and ‘hear’ are ambiguous in this respect, although context can help, as in ‘I see an apple’ vs. ‘I see a fairy.’ On Reid’s definition, to be *perceived*, an object must exist, must be sensed (by eye, ear, nose, or skin), must be experienced, and must be believed to exist (‘fixation of belief’). For example, for ‘I see a dagger before me’ to mean ‘I perceive a dagger before me,’ then my eyes must be focused on the dagger, it must be present (if not, it is an hallucination), I must experience it as a dagger, and I must believe it to be there (see also Price, 1932, *Perception*). The Common Sense School advocated a straightforward approach at the expense of a deeper analysis of the logical conditions of knowledge in epistemology and ontology; thus, the various mental categories and their properties are asserted, perhaps dogmatically, rather than derived. Such an approach might not satisfy modern philosophers, and did not satisfy philosophers subsequent to Reid, but we think it suited to the current state of psychology, in which the fundamental status of the mind, and of consciousness, remains unsettled.

Reid’s definition of perception nicely captures daily experience and seems intuitive; in particular, it excludes many of the right cases. One does not ‘perceive’ an object that is not present. If a blind person imagines an object that is actually present, he still does not perceive it visually. If a skeptic saw a real UFO landing in a field but thought it illusory, then he saw, but did not accept the reality of, the UFO: so in Reid’s terms, he did not *perceive* the UFO. If one walks downstairs while in the deeper stages of sleep, and thus one is not aware of the hallway and stairs (to judge from reports of somnambulists when abruptly woken), then one does not perceive them. Reid’s definitions invite us to not only to ask what perception actually is, but also what is it not. Are sensation and perception, for example, one and the same thing? Current researchers often run them together, in part because sensation, like perception, procures us with knowledge of the external world, or distinguish them only insofar as sensation provokes emotion. Indeed, once a needle inserted into our skin has triggered a sensation of pain, we are unlikely to forget it, and we will know in the future, whenever a needle approaches our skin again, that it will cause pain and may try to avoid it. Sensations, like perceptions, can inform the planning of adaptive behavior and allow us to cope with real-world situations. Yet sensations can arise at different levels of consciousness, during wakefulness, sleep or anesthesia, and they may be present in the absence of a real-world stimulus triggering them, as in the case of phantom limb sensations (Guéniot, 1868; Ramachandran, 1998), and thus differ from perceptions in the more demanding sense of Reid.

In Reid’s system, perception and sensation also differ *epistemologically*: that is, perception is ‘public’ in that, if I claim to perceive something that is not there, I am not perceiving, but hallucinating, whereas sensation is ‘private’; no-one can gainsay my report of pain or pleasure, no matter what external or physiological events occur. A brief quote (Reid, 1764/1977, Section XX, p. 206) illustrates his acute analysis of this usage:

“Thus, ‘I feel a pain; I see a tree’: the first denoteth a sensation, the last a perception. The grammatical analysis of both expressions is the same: for both consist of an active verb and an object. But, if we attend to things signified by these expressions, we shall find, that in the first, the distinction between the act and the object is not real but grammatical; in the second, the distinction is not only grammatical, but real. The form of the expression, ‘I feel pain,’ might seem to imply, that the feeling is something distinct from the pain felt; yet in reality, there is no distinction.”

This is in contrast to the tree, which really does exist – not just as an idea, *contra* Berkeley, but in Nature, as ‘common sense’ demands. Thus, perceptions can be tallied by accuracy, while sensations can only be rated, not scored as valid or invalid. In these respects the ‘common sense’ account cannot be reconciled with current neuroscience or psychophysics, in which perception and sensation are treated as parts of sensory continua (e.g., Chaudhuri, 2012) or treated as stages of information processing (Marr, 1982; Regan, 2000). Yet, sensation and perception are clearly distinct, as illustrated by Reid (1764/1977, Chapter 5) for touch. Stroking an object with one’s fingertips, one perceives the 3-D shape and simultaneously senses the texture or roughness; the latter, but not the former, change with the pressure and speed of the stroke. As Reid said, pain is sensed; a distant view is perceived. Sensation requires stimulation but, unlike perception, not fixation of belief about an external cause or object, so should be counted as distinct and not set on a continuum. Humphrey (1992) provides plentiful modern evidence for Reid’s claim (originally illustrated by his blind mathematician) that perception may be disassociated from sensation, as (for example) when visual perception is correct despite reversing sensation with upside-down optics, and cases of visual *agnosia* in which perception is deformed or absent while sensation remains accurate (as in cerebral achromatopsia). In contrast, a recent, superficially attractive, quotation illustrates the difficulties which occur when Reid’s distinction is ignored:

“There is a deep sense in which we all know what perception is because of our direct phenomenological acquaintance with *percepts* — the colors, shapes, and sizes (etc.) of the objects and surfaces that populate our visual experiences. Imagine looking at an apple in a supermarket, appreciating its redness (as opposed, say, to its price) and anticipating the delicious juicy sensation it will cause in the mouth when you dig your teeth into it, *that* is perception in its deepest sense” (Firestone and Scholl, 2015).

In an otherwise outstanding paper, these authors here conflate perception, sensation, and even hallucination, all of which can give rise to identical appearances.

Reid’s definition of perception does involve some difficulties. First, it is not easily applied to animal perception; one can check on the animal’s senses, but how does one know that an animal is aware of, and believes in, the food in front of it? Those of us who accept evolution and reject the creationism presumed by Reid would not want to proclaim consciousness and fixation of belief as exclusively human. Fortunately, Alex the African Grey parrot can tell us in English what he experiences and believes to be present, even including geometrical illusions: (Pepperberg, 2002). Current evidence even suggests that patterns of light are *perceived*, in the Reidian sense, not just sensed, by many birds. For example, in King Penguins, as a result of hormonal changes in the mating season, ultra-violet light (UV) becomes the source of a sensation with direct and immediate consequences for behavior (Dresp and Langley, 2006). Specifically, the UV intensity pattern reflected by the beak of a potential mate determines whether that individual is likely to be chosen as a mating partner or not, and furthermore permits accurate identification of the partner in a flock of many hundreds of birds over a considerable distance, even months later. Such an achievement in us would count as evidence for a perceptual belief. This beautiful example brings to the fore the deeper biological links between perception, sensation, hormones, and behavior.

Reid’s definition also excludes unconscious perception, which we accept, so we have had to follow 19th century practice in adding the term ‘subliminal’ to cover perceptions in which both awareness and belief are absent. Finally, the neural substrate of ‘fixation of belief’ remains to be clarified. fMRI shows that frontal lobes, subcortical structures, and cerebellum are equally involved in mental imagery and visual perception (92% voxel overlap), suggesting that images and percepts share similar access to memory, interpretation, and action control (Ganis et al., 2004). However, responses to images and percepts do differ in superior parietal lobule (the *precuneus*) and parahippocampal and fusiform gyri; and parietal differences can reflect belief status (Zaitchik et al., 2010). These results provide hints that neuroscience will eventually clarify the role of the brain in the fixation of belief interpreted as not necessarily propositional.

## Categories of Experience

As defined by Reid, perception can be distinguished from several other categories of experience. These categories form the rows of **Table 1**, and are as follows: perception, sensation, illusion, hallucination, mental image, and ‘fancy,’ the latter term being Reid’s but expanded on by S.T. Coleridge. To these we have added several modern categories: affordance, body image, subliminal percept, *ganzfeld*, and *eigengrau*. The list may prove incomplete, but it incorporates insights from neurology, psychophysics, and ecological optics. In adding and simplifying categories, we lose some of the clarity of Reid’s presentation, and we risk the inclusion of dissonant elements, but we remain true to the ‘common sense’ goal of making philosophy useful to science, in particular to experimental psychology. Rather than claiming our categories are necessary or exhaustive, we take the weaker approach of “categorical descriptivism” (Carr, 1987), which is easier to defend but is limited to describing categorical structures suggested by our thoughts, experiences, intuitions, and language.

**Table 1:** Scores.

	Properties					
Category	Mean	Distal	Object	Proximal	Aware	Belief
Perception	1.000	1	1	1	1	1
Affordance	0.800	1	0	1	1	1
Sensation	0.600	1	0	1	1	0
Illusion	0.400	0	-1	1	1	1
Body image	0.200	0	-1	1	0	1
Hallucination	0.000	-1	-1	0	1	1
Subliminal	0.000	1	0	1	-1	-1
Mental image	0.000	-1	0	1	1	-1
Ganzfeld	-0.200	-1	-1	1	1	-1
Eigengrau	-0.600	-1	-1	0	0	-1
Fancy	-1.000	-1	-1	-1	-1	-1

Each category in **Table 1** is scored on five properties; whether a distal stimulus is required; whether an external object is needed; whether proximal stimulation is needed; whether conscious awareness is required; and whether belief needs to be fixed. Four of these properties derive directly from Reid’s definition of perception (that an object must exist, must be sensed by a distal sense, must be experienced, and must be believed in); we added a fifth, whether a proximal stimulus is needed, as this is not implied by the others. The properties are complete to the extent that they define all the ways experience and reality can inter-relate structurally – that is, without regard to specific content.

We scored each property using a ternary system, as +1 if it held true of a category, -1 if it did not, and 0 if it was irrelevant or ambiguous. Binarizing by assigning +1 to truth and -1 to falsity is standard, but using 0 (rather than, say, +0.2 or -0.3) is valid only on the assumption that degrees of uncertainty are unsystematic and average to zero. All scores have the same direction in terms of realism, from perception (all +1) to fancy (all -1), so the mean score indexes reality. Between perception and fancy lie the intermediate categories, which we now characterize in the order shown in **Table 1** from the most to the least reality-oriented. The next section justifies our scores for each of the categories in turn.

### Perception

As already discussed, we take Reid’s definition of perception literally: to be perceived, an object must exist, must be experienced^3^, must be believed to exist, and in addition, there must be a proximal stimulation to a distal sense (eyes, ears, touch, and smell). Thus percepts are scored as +1 on every property in **Table 1**. We note that perceptions of absence, such as “I perceive no elephant in my room,’ are not included in this definition. Absence is redundant, in that the numerous possible objects whose presence is to be denied are implied by what I *do* see, given that objects are opaque. Here, we follow Reid: “The more obvious conclusions inferred by reason from our perceptions constitute what we call *common understanding*” (Reid, 1764/1977, Chapter 6); that there are no elephants illustrates a common understanding, not a perception.

### Affordance

An ‘affordance’ is a feature of the physical environment that permits (‘affords’) a behavior, such as the ground being flat enough to permit running, as defined by Gibson (1979). This concept has proven so useful (see Carello and Turvey, 2003, and many others) that we think it needs consideration. Affordances requires proximal and distal stimuli (both properties scored +1 in **Table 1**), but not necessarily visual objects (scored 0), as affordances can also specify landscape, air, lakes or sea. In Gibson’s system, affordances are detected by picking out invariants in the optic array, such as the ‘nesting’ of visual angles. Since the information specifying the affordance must be actively attended to affect action (Gibson, 1979), we score ‘awareness’ and ‘belief’ as +1. Affordance is otherwise similar to Perception in **Table 1**, but they also differ in emphasizing environmental information and action (affordance) vs. neural processing and awareness (perception). Thus affordance and perception are not equivalent – and neither are equivalent to action.

### Sensation

A ‘sensation’ is a mental event requiring processing activity in a sense organ or at higher levels of brain integration, as in the case of phantom limb sensations. Sensation requires a sense organ to register a proximal stimulus (score +1), as when one senses light or sound, or one feels bodily pleasure. In normal seeing and hearing, proximal stimulation is nearly always caused by distal stimuli (score +1), although one can also see stars when one rubs one’s eyes. Following Reid, sensations do not require that their origins be interpreted; there is no necessity that an object exist, or nor that belief be fixed (scores 0), though they may be. Because sensation is so tied to the sensory nerves, it can be defined medically as the response in the brain to neural activity originating in the sense organ, but here, the phenomenal nature of the sensation, not the neural channel communicating it, is primary. A sensation is first and foremost an experience – it requires the activity of the sensory organ, but cannot be defined by it^4^.

### Illusion

An illusion in psychology is a mistaken percept, one that one may know is wrong but cannot correct – being ‘cognitively impenetrable.’ Classic examples include the Mueller-Lyer, Poggendorf, and wagon-wheel illusions; modern illusions include a host of motion-generated illusory percepts. In all cases, one is aware of the object, but it is misinterpreted, so we score +1 for awareness and -1 for object. Indeed, illusions typically derive from incomplete stimulation, as pointed out by Gibson (1979). Illusions produce beliefs (score +1), false as they are. Illusions require proximal stimuli – they are not hallucinations (score +1). Distal stimuli are commonly present but are not required (score 0), as in the cutaneous ‘rabbit’ (Geldard and Sherrick, 1972). Proximal or distal stimuli must clearly differ from their illusory interpretations^5^.

### Body Image

A body image can be kinesthetic, tactile, motor, or some combination of these. Body images are generally accurate enough to support behavior, as in running through a narrow opening. A body image differs from a visual image of one’s body in being partly motor. False body images exist as, for example, in *anorexia nervosa* where people perceive themselves as unrealistically fat, or in schizophrenia where they may perceive parts of their body as distorted (*dysmorphophobia*: see Cororve and Gleaves, 2001). After surgery or amputation, body images or perceptions of one’s body “as it was before” may occur, as demonstrated for example by the famous phantom limb observations where patients describe sensations including strong pain in amputated limbs as if they were still part of the body (e.g., Guéniot, 1868; Ramachandran, 1998). In extreme cases, the body image is sufficiently dysfunctional to impede simple activities, such as walking through a narrow opening or picking up a cup. How should we score body images? Clearly there must be a proximal stimulus, i.e., an afferent input to the brain, and there must be a belief that the body (or part of it) it exists and has such-and-such a form (both scored as +1). There is no external object (score -1), so body images are surely not perceptions. We suspect that the body image may be influenced by distal stimuli, even if these are not essential, as demonstrated by Witkin’s ‘tilted room’ which distorts the sense of being upright (score 0). The body image may be bought to awareness, as during an activity like dressing, but this is also not essential as it can fade from view without ceasing to affect behavior (score 0).

### Hallucination

A ‘hallucination’ is a form of false percept where the subject is aware of, and his belief is fixated on, an event that appears to take place in the real world (both score +1), but distal stimulation and real-world objects are absent (both score -1). An example is the man who ‘mistook his wife for a hat.’ Hallucinations do not have to, but may involve proximal stimuli (score 0), since such stimulation may also arise within the brain (Grossberg, 2000; Allen et al., 2008). There is an enormous literature on hallucinations, their origins, and sub-types, which might well lead to sub-categories in a future development, but given the five properties of interest here, we characterize them *en masse* by the scores in **Table 1**.

### Subliminal Perception

A ‘subliminal percept’ requires both a proximal and distal stimuli stimulus (scores of +1), and an interpretation of the distal stimuli at some level of neural processing, but one that paradoxically escapes awareness and therefore, fixation of belief (each scored -1). An external object may, but is not required to, create a subliminal percept (score 0). Despite demonstrable effects of subliminal on subsequent percepts, subjects claim to see nothing, as for example in metacontrast masking (Scharlau and Neumann, 2007), in continuous flash suppression (Lin and He, 2007), and in human blind sight (Weiskrantz, 2009). Since subliminal perception exists, and is not simply an artifact of criterion-shifting, it requires its own category^6^.

### Mental Images

A ‘mental image’ is a sight or sound that reproduces an object of perception, or combines such objects in an agglutinative fashion, from memory – thus, in the absence of a distal stimulus (score -1). There is no belief in the external reality of the image (score -1): if one believed in it, one would be hallucinating. There needs to be some proximal stimulus, although it may be distant from the resulting mental image, as when the mere feel of a carpet triggers an image of the carpet in front of a fireplace with a cat asleep on it (score +1), to account for the ‘concrete’ nature of the image (images that have no proximal stimulus at all are classified as ‘fancy’). In Reid’s view, Hume (1739/1992) was driven by a false metaphysics to assume that images and percepts differ only in vividness. Indeed, images and percepts generate somewhat similar EEGs (LaBerge, 1990) and fMRI scans (Ganis et al., 2004). However, psychophysics shows that the spatial and temporal effects of images and percepts are quite different (Arterberry et al., 2002). In terms of the properties we take as defining, images and percepts are indeed far from equivalent^7^, as indicated in **Table 1**.

### Ganzfeld and Eigengrau

A *Ganzfeld* is a uniform fog, in which visual stimulation by light is too even for distinct objects to be visible. The auditory equivalent would be white or pink noise; in both cases, there is a proximal stimulus (score +1). The *Eigengrau* corresponds to the internal level of uniform stimulation generated in the eye (or, analogously, in the ear), independent of proximal stimulation (scored 0). A distal stimulus, an external object, and belief in external reality are contra-indicated for both categories (scored -1). One is aware of a *Ganzfeld* (score 1), but not necessarily of the *Eigengrau* (score 0). Both categories may seem esoteric, but have proven important in visual science since Fechner, and so have a place in **Table 1**.

### Imagination or ‘Fancy’

At an opposite extreme from perception is ‘fancy,’ or pure imagination, in which the mind invents visions or sounds unrestricted by reality that can be summoned or dismissed at will (Reid, 1786). Such fancies may be bound by the colors and sounds that exist in Nature or in artifice – but their combinations, like abstract painting or musical symphonies, go far beyond mere concatenation, eliciting complex and often novel sensory experiences. A fancy is believed to be internal to the mind, or is thought to descend from God as Beethoven believed, but is not from dependent on an external object or stimulus (score -1). No form of stimulation, proximal or distal, is involved, and so awareness of external stimuli is ruled out (score -1). Imagination is a creative process with but limited input from memory or perception, so for example a portrait or landscape painter who simply paints what he sees would not count as creating a ‘fancy,’ no matter how delicate or novel his method, whereas an abstract painter might so count. That fancy scores -1 on each property makes it the opposite of perception in the scheme of **Table 1**.

### What Can We Learn from Analyzing the Scores?

As already stated, we scored each property in the same direction, with 1 representing reality and -1 fancy. The use of a scoring scheme for mental categories is novel and admittedly debatable, and using a ternary score (+1, 0, -1) may improve on a simple binary (True/False) logic but still ignores degrees of uncertainty. However, a score sheet such as **Table 1** illuminates a useful vocabulary of mental states, and perhaps offer up some new insights.

Given the scores in **Table 1**, one may look for numerical patterns within them. **Table 2** provides an index of how different the categories are from each other. Diagonal elements in this Table are all zero since categories do not differ from themselves, and the upper triangle is redundant, being a reflection of the lower one. Each entry is the root-mean square (RMS), that is, the square root of the mean squared differences between categories, the mean being taken over the five properties. RMS values are scaled to lie between 0 and 1 by dividing by the maximum possible value. The initial squaring ensures that positive and negative differences count equally instead of canceling. To illustrate from the first column, since perception and affordance differ little, RMS is small (0.22); since perception and mental imagery differ more, the index is higher (0.67). Perception and fancy differ maximally (1.0). The RMS values point toward a more nuanced conceptual landscape than provided by a simple same/different analysis.

**Table 2:** RMS Differences across properties.

Category	Pcpt	Afford	Sensat	Illus	Body	Halluc	Sublim	Mental	Ganz	Eigen
Perception	0.00									
Affordance	0.22	0.00								
Sensation	0.32	0.22	0.00							
Illusion	0.50	0.32	0.39	0.00						
Body image	0.55	0.39	0.45	0.22	0.00					
Hallucination	0.67	0.55	0.59	0.32	0.39	0.00				
Subliminal	0.67	0.63	0.50	0.71	0.59	0.84	0.00			
Mental image	0.67	0.63	0.50	0.55	0.59	0.55	0.63	0.00		
Ganzfeld	0.77	0.67	0.55	0.50	0.55	0.50	0.67	0.22	0.00	
Eigengrau	0.84	0.74	0.63	0.59	0.55	0.50	0.59	0.39	0.32	0.00
Fancy	1.00	0.92	0.84	0.81	0.71	0.67	0.67	0.67	0.63	0.32

**FIGURE 1:**
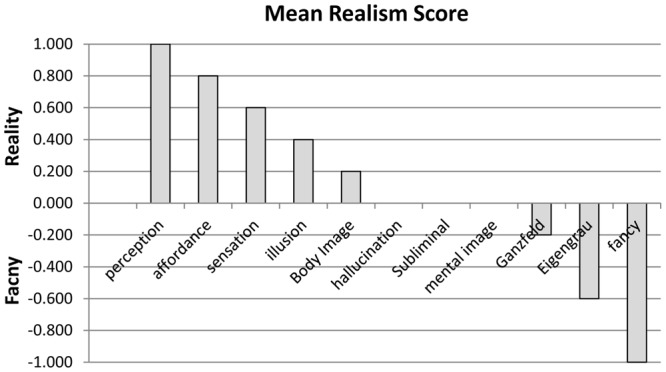
Mean realism per category.

Subsequent Tables present more advanced statistical comparisons. **Table 3** compares the categories, not in absolute units like RMS, but rather in terms of variability, since scores that differ absolutely may nevertheless co-vary: for example, each Ganzfeld score equals 1+ twice the corresponding Eigengrau score. **Table 3** is the variance/co-variance matrix, in which diagonal elements (in italics) are variances and values below the diagonal are co-variances; perception and fancy, being constant with no variance, are excluded. **Table 4** extracts from **Table 3** the proportion of variance in each category ‘accounted for’ by each other category, as indexed by *r*^2^. This is the square of Pearson’s correlation coefficient, *r*, and equals the ratio of the covariance to the geometrical mean of the two category variances. Using this metric, which is standard in statistics, the variance accounted for lies between 0% (no relation) and 100% (entirely predictive). **Table 4** shows how much the categories predict each other, *r*^2^ being 100% between *Ganzfeld* and *Eigengrau* and greater than 40% for 7 other pairs (bold-face in **Table 4**). In the remaining cases *r*^2^ is small, indicating that these categories approach statistical independence (*r* = 0). To the extent independence holds, the mean score for each category provides a measure for that category which is uncontaminated by the other categories. Mean scores are provided in **Table 1** and plotted against category in **Figure 1**. These means agree well with the authors’ intuitions concerning realism; the higher the mean score, the more reality-based the category.

**Table 3:** Variance/co-variance matrix.

Category	Afford	Sensat	Illusi	Body	Halluci	Sublim	Mental	Ganz	Eigen
Perception = 0									
Affordance	*0.20*								
Sensation	0.15	*0.30*							
Illusion	0.35	0.20	*0.80*						
Body image	0.30	0.10	**0.65**	*0.70*					
Hallucination	0.25	0.00	**0.75**	0.50	*1.00*				
Subliminal	0.00	0.25	-0.25	0.00	-**0.75**	*1.00*			
Mental image	0.00	0.25	0.25	0.00	0.25	0.00	*1.00*		
*Ganzfeld*	0.20	0.40	**0.60**	0.30	0.50	0.00	**1.00**	*1.20*	
*Eigengrau*	0.10	0.20	0.30	0.15	0.25	0.00	0.50	**0.60**	*0.30*

*Values for perception and fancy are zero. Variances are italicized; covariances above 0.6 or below -0.6 are in bold.*

**Table 4:** Variance in each category accounted for (***r*^2^**) by each other category.

*r*^2^	Afford	Sens	Illus	Body	Hallu	Subl	MentIm
Sensation	0.38						
Illusion	**0.77**	0.17					
Body image	**0.64**	0.05	**0.75**				
Hallucination	0.31	0.00	**0.70**	0.36			
Subliminal	0.00	0.21	0.08	0.00	**0.56**		
Mental image	0.00	0.21	0.08	0.00	0.06	0.00	
Ganz/Eigen	0.17	**0.44**	0.38	0.11	0.21	0.00	**0.83**

*Perception and fancy have no variance and are excluded; ganzfeld and eigengrau are perfectly correlated (r = 1) and are tabulated together. Bold-face indicates high values (those over 40%).*

Similarly, one can also ask if the five properties (rather than the categories) are distinct or not. This can be answered by calculating the variance in each property accounted for by each other property, again expressed as *r*^2^, taken across all 11 categories. **Table 5** shows how the properties inter-relate; most of the *r*^2^-values are satisfyingly low, implying near-independence of the properties, with only one pair (object and distal) with an *r*^2^ over 40%.

**Table 5:** Variance in each property accounted (*r*^2^) by each other property.

	Distal	Object	Proximal	Aware	Belief
Distal	1.000				
Object	**0.491**	1.000			
Proximal	0.326	0.222	1.000		
Aware	0.003	0.051	0.260	1.000	
Belief	0.180	0.021	0.088	0.238	1.000

*The bold-face value is over 40%; all other values are low.*

Our five properties are consistent and fairly independent, but are they complete? This is a much more difficult question to answer. We have implicitly adopted a ‘constructivist’ view of perception (Norman, 2002), in which elements of the world (distal stimuli, objects) which stimulate sensory organs give rise to awareness and to decisions about what is ‘out there,’ fixing the beliefs necessary to guide behavior. The five properties in **Table 1** index the essential elements in this process, and so are reasonably complete in this sense. However, **Table 1** ignores interactions among categories – Wagner could compose music (fancy) only when he could touch velvet (sensation) – and ignores the role of feedback, i.e., the effects of attention, expectation, and memory on perception. **Table 1** also says nothing about how action controls on-going environmental information pick-up (Gibson, 1979). Can we account for these lacunae? Norman (2002) distinguished constructive from ecological processes, the former making inferences beyond the elementary sensations (‘indirect’ perception) and the latter processing the incoming sensory stream directly (‘direct’ perception). Norman’s two systems theory associated constructive processes with the brain’s ventral system for visual object recognition and knowledge and ecological processes with the brain’s dorsal system for acquiring scene layout and optic flow to control bodily actions. We suggest by analogy with vision that auditory recognition, that is, interpreting meaningful sounds, such as speech, specific sounds, and bird song, may also count as constructive, but not the perception of auditory layout variables such as sound volume, distance, profile analysis, and echo suppression. Constructivism could also apply to touch and smell as distal systems giving us interpretable information about external objects (see Reid, 1786, Essay 2, Chapter 16), – the stink of an abattoir, the soft feel of corduroy, the touch of a hand – but not to the ecological information such as the breathability of the air or the pressure on one’s feet (needed for activities such as running or balancing). To the extent that the two systems survive empirical testing and show up in distinct brain anatomies, ecological and object information differ fundamentally and **Table 1** will only apply in any detail to the latter.

Without some classification system, even as crude as that given in **Table 1**, the fundamental categories of mental experience will be necessarily confused. For example, in their review, Firestone and Scholl (2015) classify six frequent methodological errors in the literature claiming ‘top-down’ or cognitive effects on perception, errors which they rightly say must be avoided if top-down influences on perception are to be firmly established. However, their definition of perception in terms of appearance and not in terms of veridicality, *contra* Reid, weakens their conclusions, we would contend; only if fixation of belief is included in the very definition of perception can appropriate empirical test cases be analyzed.

## Color as a Test Case

Color provides an interesting, if complex, test case for our classification. Color is just one of many attributes, such as texture, shape, and size, but one that has been intensively investigated for more than a century by experimenters, and before that by philosophers such as Reid (1764/1977, Chapter VI). Color categories provide a natural interpretation of the color experience, one which is ‘public’ – that is, can be verified by others. Following Reid, if one ‘perceives’ a yellow sun, it cannot be orange or gray; likewise, if one perceives a red apple, it cannot be a green apple or a red banana. Interestingly, all trichromats agree that wavelengths around 580 nm are ‘yellow,’ despite vast differences in retinal signaling due to vast differences in the relative numbers of the three classes of cones across individuals and, within individuals, across retinal eccentricity. Here, differences in the sensory organ (the retina) do not produce commensurate differences in sensation. Indeed, narrow-band spectral lights isolated in a small aperture will be placed in the same order by every trichromat, to a tolerance of a few nanometers, so color perception is to this extent veridical even when disassociated from known references or shapes. This does not imply identity of experience or appearance across individuals; the colors of narrow-band lights could be inverted (Block, 1990: my short-wavelengths seen as ‘red’ not ‘blue’) or down-shifted (the neural correlate of ‘yellow’ in my brain, if transported to your brain, might appear ‘green’ to you), but still, colors are not randomized - color *order* is preserved. True, every trichromat will see some combination of red and green lights as matching a narrow-band yellow light, a physically incorrect response. Since wavelengths sum in the cones, with a limited number of cone types, metamers, i.e., physically different lights that generate identical sensations, must exist. This has been taken as a major limitation for color vision, but, in turns out, metamers are actually rare in natural scenes (Foster et al., 2007). Given an adequate sensory signal, *color constancy* provides a test of color as a ‘perception.’ To the extent that the color of an object can be identified independently of the chromaticity of the illumination striking the object, the goal of perception is being met, as clarified by Reid (1764/1977, IV); hence, to the extent that the color and brightness of the illuminating light affect the color appearance of the object, perception has failed. Since the light at the eye is a product of illumination and reflectance, perceiving the object color requires discounting the illuminant. Most individuals can perceive and recall the colors of familiar objects (such as their own handbag) correctly, no matter what the illumination (Weiss et al., 2015). Even when the ‘object’ is just a colored square surrounded by other squares, as in painting by Mondrian, most individuals can report the object color (showing reasonable color constancy). They can also report the light at the eye coming from the same colored square without discounting the illuminant (Arend and Reeves, 1986), a distinction we characterized as a ‘paper match’ task or as a ‘direct match’ task, the one being perceptual and the other, sensory, unaware that Reid had already pointed out the distinction (and that artists were skilled in both tasks).

Color vision also provides a contrast between sensation/perception and affordance (Gibson, 1979, Chapter 6). Color can afford specific actions, such as walking on a wet or soft surface vs. jumping over it, or picking a ripe fruit, or knowing the time of day from the skylight. In each case, the information provided by the environment is ‘nested,’ object colors being located within local environments that are in turn bathed in the illuminating sky-light. Mechanisms have evolved for extracting information with survival value, by taking advantage of nesting, but information not relevant for survival is ignored. (Thus, there need be no fully general perceptual systems, possibly Gibson’s greatest insight). The red–yellow–green categorical structure affords fruit picking, for example (Sumner and Mollon, 2003), whereas the rainbow colors afford no actions but are purely sensory, merely giving pleasure. Thus the vast body of color research devoted to understanding how wavelength is encoded by the eye and how colors are perceived in the cortex of the brain has almost nothing to say about affordance. Only an analysis of the visual environment can tell us what color can do for us, what actions it can or cannot afford. Given the need for plants and animals to both display (to on-specifics) and to hide (from predators), Pinna and Reeves (2015) made a recent attempt, arguing that the visual purpose of color is to promote the emergence of the whole, to support a part–whole organization in which components reciprocally enhance each other, and to reveal fragments and hide the whole (camouflage). They noted that these processes have been revealed in human psychophysics but not in animals, so their evolution is as yet unknown.

### The Peculiar Status of ‘Perceptual Illusions’

In Reid’s (1786), system perception and illusion are clearly distinct. What sense, then, can one make of ‘perceptual illusions’? To be ‘perceptual,’ an illusion must reveal a true state of Nature, which sounds contradictory but may be illustrated by looking steadily at a sheet of white paper placed on a gray background. The paper is, and is perceived to be, uniform white. Nevertheless, due to receptor adaptation, the center of the white area generates exactly the same retinal responses as the dark gray surround. The receptors signal only that the light level increases as the eye traverses a dark-to-light edge, generating a thin surrounding frame (thus if eye movements are stopped with wax, and the head clamped, the percept of the white paper disappears and all seems gray: Mach, 1914). The uniform white experience is generated in the brain given no indication to the contrary, that is, *not* seeing different light levels within the white area. This lack of sensation is the cause of the visual brain ‘filling-in’ from the white-coded edges into the center, giving rise to an illusory white percept. Yet, the illusory white is true to Nature, so the outcome is a percept, in Reidian terms. The illusory nature of the filling-in percept can be seen clearly with bichromatic outlines. An outer line of purple with an inner line of orange, enclosing a white area, creates an illusory fill-in color of orange over the entire extent of the drawing; this is Pinna’s now famous ‘watercolor’ effect (Pinna et al., 2001). In the ‘back-lighting’ effect, an illusory halo is seen surrounding a figure due to its bi-chromatic edges, one that creates a sense of volume (Pinna and Reeves, 2006). But in the case of white paper on a gray field, it *really is* uniform white; the illusion generated in the visual brain corresponds to reality, and is seen as such. Hence, we might consider ‘perceptual illusion’ as a category. Indeed, almost all of the visual field is a perceptual illusion (and there are analogies in all other sensory modalities) since the brain must correct sensations that have been distorted by the sensory organ (e.g., the optics of the eye and retinal processes such as blood vessels which block light), but to the extent that the corrections are good, the final outcome will correspond to reality. Therefore, perceptual illusions are ultimately in the same general category as percepts, and should best be thought of as a sub-category of perception. They do not lead to delusions but rather permit the perceptual system to produce to best perceptual hypothesis possible when stimuli are incomplete and ambiguous (this is the popular Bayesian hypothesis, when applied to perception). Remaining distortions like the water-color effect, though remarkable, are generally of small magnitude and probably do not affect adaptive visual behavior.

### The Particular Status of Moods and Emotion

In Reid’s common-sense philosophy, perception, sensation, and emotion are distinct; perception provides knowledge, whereas sensation can trigger emotion and disrupt knowledge. Modern psychology has no overarching theory of emotion/perception interactions, but it does have an enormous accumulation of examples of such interactions, all ignored in **Table 1**. Thus darker colors illicit somber moods and lighter colors more cheerful ones; saturated colors (red) elicit different moods than desaturated ones (pink). Mental associations between specific colors and specific perceptions are readily made, as a pool of red color on the tarmac may be perceived as blood even when it is paint. Interactions of this sort speak to a larger frame of reference in which emotion and feeling tone are included, as well as sensation, action, imagination, and perception.

## Conclusion and Perspectives

Ideally our analysis of Reid’s philosophical approach to perception remains true to the Common Sense School’s goal of establishing useful categories. A key outcome of our analysis is that the processes by which we perceive stimuli can be grouped into different categories with different scores for various properties, according to Reid’s own definition of perception. We recognize that these categories and properties may need further expansion to go beyond vision and help us to better understand the other senses and especially multimodal perception, that is to characterize the conditions under which stimuli combine effectively. This may eventually lead us to redefine Reid’s idea of ‘perception’ in terms of the observable product of signal reception, integration, and processing in a larger realm than just the visual one on which our analysis was focused. Whether or not ‘multi-perceptual’ and ‘multisensory’ systems will turn out to differ in the ways that we claim visual perception and sensation to differ will become clearer as multisensory research progresses. Most important, clarifying terms should both aid empirical research and facilitate communication between philosophers and scientists. Finally, we rather hesitantly have put forward an example of what me might call ‘computational philosophy,’ in which a perhaps simplistic ternary scoring system permits statistical analysis, with the hope that others may take advantage of this approach when appropriate for other philosophically inspired treatments of mental states.

## Author Contributions

AR wrote the initial paper. BD-L provided philosophical insights and corrections.

## Conflict of Interest Statement

The authors declare that the research was conducted in the absence of any commercial or financial relationships that could be construed as a potential conflict of interest.

## Footnotes

1 This is not the place for a discussion of Reid’s epistemology, which would take us too far afield and is already well covered in the literature (see, e.g., Lehrer, 1989). However, an unfamiliar reader should know that he was a student of Hume who eventually rejected his master’s thesis that mental contents depend solely on *sensations*, *images* (representations of sensations) and a few laws of association. Reid did accept Berkeley and Hume’s point that “We cannot, by reasoning from our sensations, collect the existence of bodies at all, far less, any of their qualities” (Reid, 1764/1977, p. 7). Nevertheless, he rejected Berkeley’s claim that ‘nothing can be like an idea but an idea,’ as this implied that ideas cannot resemble features of the world, and concluded that “all the labored arguments of the skeptical philosophy against a material world, and against the existence of everything but impressions and ideas, proceed upon a false hypothesis” (p. 43). Reid’s knock-down argument is that to be consistent, a skeptic who distrusts his sensations (that imply an external world) should equally well distrust his reason (that concludes the world may not exist) and his memory (that implies past existence), and therefore should abandon any attempt to understand either mind or nature. Rather, a true philosophy should accept that “the evidence of sense, the evidence of memory, and the evidence of the necessary relation of things, are all distinct and original kinds of evidence, equally grounded in our constitution” and critically, “To reason against any of these kinds of evidence is absurd; nay, to reason for them is also absurd. They are first principles; and as such fall not within the province of reason, but of common sense” (Reid, 1764/1977, p. 122). Instead, Reid supported a form of nativism in which the mental faculties (sensation, reason, etc.) are either present at birth or unfold over time, and are modified but not generated by, experience.

3 A technical point arises here. We take ‘must be experienced’ to cover three important sub-cases in visual perception, all distinguished by the Gestaltists; perception of the whole, modal completion, and a-modal completion. Perception of the whole occurs when an object is fully visible; modal and a-modal completion may occur when objects are seen behind partial occluders, and a-modal when they go out of sight. In the modal case, the brain completes or fills in the missing information such that the entire object is experienced consciously, whereas in the a-modal case, the perceiver sees and experiences only a part, but apprehends the whole. To illustrate, place two pencils on a table with one crossing over the other; the top one is seen wholly, while the bottom one is seen modally if one fixates far from the pencils and a-modally if one fixates the cross point. Such completions illustrate ‘apperception’ (Herbart, 1816), that is, knowledge of an object that goes beyond the immediately present but is non-verbal, as novel, un-named objects are equally subject to completion.

4 Purely physiological and behavioral approaches to sensation, such as in the vast majority of research papers in vision and audition over the last few decades, as summarized in the well-regarded textbook of Chaudhuri (2012), by-pass the unexplained element of consciousness in order to concentrate on known sensory and neural structures. This is all very well for a purely medical approach, but is becoming out-dated in failing to acknowledge the role of consciousness (e.g., Dehaene, 2014).

5 For example, punctate stimuli may be experienced as a continuously flowing stream, as when a sequence of consecutive notes is experienced as a ‘run,’ or a sequence of animated cartoons from the 1910s gives rise to visual apparent motion despite appearing jerky. Illusions may be optical, as when objects reflect light in a specific way, suggesting perception of physical matter or objects (water, gold, *et alia*) that are not present. To the extent that an illusion is purely optical, as, for example, a mirage that can be captured by a camera, it needs no explanation in terms of the mind, and would not appear in **Table 1**. However, some illusions straddle the boundary between optical and perceptual, as when a glittery surface appears wet. We have not included a sub-category for this case, although a more complete taxonomy of illusions would do so (Gregory, 1997).

6 Reid did not, although he noted that some appearances may be so fleeting as to be unrecognizable. Indeed, referring to sight: “If we pay proper attention to how our mind operates in our use of this faculty, we shall become aware that the visible appearance of objects is something we hardly ever notice” (Reid, 1764/1977, p. 51). His examples include the changes of color and perspective that occur when changing one’s viewpoint of an object perceived to remain constant; the changing views lead to fleeting appearances that are almost instantly transformed into stable percepts and are normally invisible. However, Reid claimed these appearances are not invisible to artists, being fleeting rather than absent, and in this respect, they can be counted as percepts – and so differ from metacontrast and flash suppression, which remain subliminal no matter how much training, feedback, and practice is given.

7 Hume’s theory required that “sensation, memory, belief, and imagination, when they have the same object, are only different degrees of strength and liveliness in the idea” (Reid, 1764/1977, p. 15), a proposal that Reid found non-sensical. Rather: “Sensation and memory therefore are simple, original, and perfectly distinct operations of the mind, and both are original generators of belief. Imagination is distinct from both, but does not generate belief.” Importantly, “Sensation implies the present existence of its object; memory its past existence; but imagination views its object nakedly, without any belief in its existence or its non-existence” (p. 14). For Reid, these arguments are critical in epistemology as they undermine Humean skepticism and point toward the existence of distinct, innate, human mental faculties; but here, we simply take his characterizations as evidence for distinct categories.
